# Optimizing intraoperative conditions in patients undergoing elective colorectal surgery to prevent anastomotic leakage: SmartCheck study

**DOI:** 10.1093/bjs/znaf236

**Published:** 2025-12-01

**Authors:** Taskforce Anastomotic Leakage, Anne de Wit, Daitlin E Huisman, Boukje T Bootsma, Geert Kazemier, Jennifer M J Schreinemakers, Bas Frietman, Lindsey de Nes, Freek Daams

## Introduction

Initiatives aimed at preventing colorectal anastomotic leakage (CAL) have expanded in recent years. Efforts targeting optimization of preoperative and intraoperative conditions, such as enhanced recovery after surgery (ERAS) protocols and prehabilitation programmes, have demonstrated beneficial effects in patients undergoing colorectal surgery^1–3^. More recently, an additional preventive strategy has been proposed, focused on mitigating modifiable CAL risk factors^4,5^. This strategy involves implementation of an additional intraoperative timeout moment to assess exposure to CAL risk factors, combined with a six-part enhanced care bundle, designed to optimize these risk factors and thereby improve intraoperative conditions. In a controlled study setting, this optimization strategy was associated with a significantly lower CAL rate compared with non-optimized patients (6.2% *versus* 8.6%; OR 1.52; *P* = 0.045)^5^.

Outcomes observed in controlled study settings often exceed those seen in routine clinical practice^6,7^. For example, studies on ERAS protocol implementation report compliance rates of <70% in real-world clinical settings^8,9^. A similar limitation may apply to the abovementioned study targeting CAL risk factor reduction, during which continuous support throughout the entire care pathway and external oversight of compliance were applied.

Implementing the enhanced care bundle without supervision and feedback would more closely reflect real-world clinical practice, where sustained behavioural change must occur without ongoing external support. Using an intraoperative checklist alone may provide a feasible and effective strategy for mitigating modifiable risk factors by encouraging the surgical team’s self-learning capacity. This pragmatic approach could promote the sustainable application of research-driven improvements, while maximizing effectiveness. However, it remains unclear whether this approach successfully reduces exposure to modifiable CAL risk factors. The primary aim of this study was to assess whether intraoperative optimization could be achieved solely through using an intraoperative checklist. The secondary aim was to evaluate the effect of this optimization strategy on complications, CAL, and mortality. It was hypothesized that the sole implementation of an intraoperative checklist, without external supervision or feedback, would be a feasible and effective method for reducing exposure to modifiable CAL risk factors in real-world clinical practice.

## Methods

### Study design

This was a prospective, non-randomized, comparative cohort study conducted between September 2021 and December 2024 in one Italian hospital and nine Dutch hospitals. The study was approved by the Medical Ethics Review Committee of Amsterdam UMC (2020.0608) and all participating hospitals. It was declared exempt from the Medical Research Involving Human Subjects Act (WMO). This study adhered to the principles of the Declaration of Helsinki. Written informed consent was obtained from participants for data analysis and publication. This study was pre-registered at http://www.clinicaltrials.gov (NCT05250882; 22 February 2022).

### Patients

All consecutive adult patients undergoing elective colorectal surgery involving the creation of a coloanal or colorectal anastomosis were included. Both benign and malignant surgical indications were investigated and diverting stoma formation was permitted. Exclusion criteria included emergency surgery, age <18 years, absence of an anastomosis, and inability to provide informed consent.

### Procedures

All clinicians involved in the care of patients undergoing colorectal surgery were provided with the scientific evidence regarding the six modifiable CAL risk factors (anaemia, hyperglycaemia, hypothermia, incorrect antibiotic prophylaxis timing, vasopressor administration, and epidural use), along with their corresponding ORs^4^. Exposure to these risk factors was scored using an intraoperative checklist. The checklist was completed during a single additional timeout moment, conducted just before creation of the anastomosis, in a collaboration between the surgical and the anaesthetic teams.

**Fig. 1:**
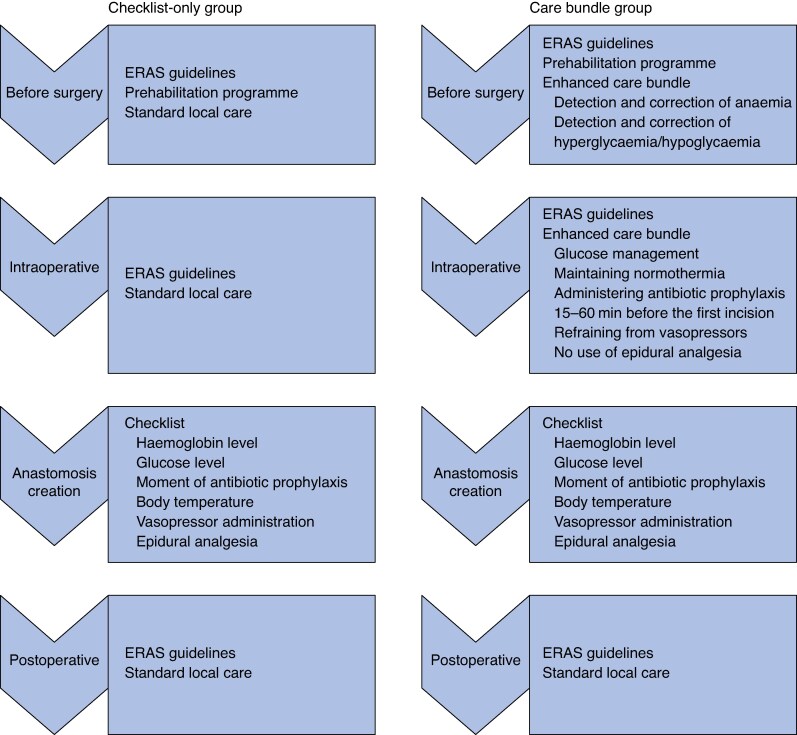
Study groups ERAS, enhanced recovery after surgery.

Patients were divided into two groups: a checklist-only group and a care bundle group (*Fig. 1*). Study group allocation was determined at hospital level, with each hospital employing only a single optimization strategy. For patients enrolled in the checklist-only group, the only study intervention was assessment of intraoperative risk factors using the checklist before creation of the anastomosis. Preoperative and intraoperative interventions aimed at preventing or detecting early exposure to these risk factors were not formally integrated into the local care pathways. After completion of the checklist, no predefined interventions were mandated to mitigate the identified risk factors. Additionally, no recommendations were provided regarding stoma construction based on the checklist findings. All clinical decisions made after checklist completion were left entirely to the discretion of the surgical and anaesthetic teams.

In contrast, for patients enrolled in the care bundle group, an enhanced care bundle was added to the intraoperative checklist and incorporated into the local care pathways. This bundle comprised targeted interventions aimed at optimizing the six modifiable CAL risk factors. Furthermore, clinicians received regular interim feedback on their compliance with the enhanced care bundle during the study interval.

In both groups, preoperative and intraoperative care was otherwise provided according to standard clinical practice, with ERAS guidelines implemented before study initiation^10^. Routine intraoperative bowel perfusion testing using indocyanine green was performed during rectal surgery, while its use during colonic resections was at the discretion of the lead surgeon based on intraoperative judgement. Before surgery, all patients were scheduled to participate in prehabilitation programmes^11^.

### Outcomes

The primary outcome was the intraoperative condition, quantified as the number of modifiable CAL risk factors present during the intraoperative checklist (expressed as mean and ≥3). Intraoperative anaemia was defined as haemoglobin levels <7.5 mmol/l (12.1 g/dl) in female patients and <8.0 mmol/l (12.9 g/dl) in male patients. Blood glucose levels >10 mmol/l (16.1 g/dl) or <4 mmol/l (6.4 g/dl) and body temperatures <36.5°C were regarded as abnormal. Incorrect antibiotic prophylaxis was administration outside the window of 15–60 min before the first incision. Additionally, vasopressor administration in euvolemic patients and epidural analgesia in minimally invasive surgery were discouraged (*Table 1*)^4,5^.

**Table 1:** Modifiable CAL risk factors

Modifiable risk factor	Definition
Anaemia	Haemoglobin level: <7.5 mmol/l (12.1 g/dl) in female patients and <8.0 mmol/l (12.9 g/dl) in male patients
Hyperglycaemia	Blood glucose level: >10 mmol/l (16.1 g/dl) or <4 mmol/l (6.4 g/dl)
Hypothermia	Body temperature: <36.5°C
Incorrect antibiotic prophylaxis	Administration: <15 or >60 min before the first incision
Vasopressor administration	Administration: in euvolemic patients
Epidural analgesia	Applied: in minimally invasive surgery

CAL, colorectal anastomotic leak.

Secondary outcomes were 90-day CAL and overall complication rates. Postoperative complications included CAL, ileus, gastroparesis, wound infection, bleeding, pneumonia, urinary tract infection, thromboembolic events, atrial fibrillation, mortality, and other unspecified complications. CAL was defined according to the criteria of Reisinger *et al*.^12^ and graded according to the International Study Group of Rectal Cancer (ISREC) classification^13^. Overall complication severity was evaluated using the Clavien–Dindo classification^14^.

A comprehensive assessment of the intraoperative condition was conducted using a checklist consisting of three domains: general condition (haemoglobin, glucose, moment of antibiotic prophylaxis, body temperature), local perfusion and oxygenation (blood loss, blood transfusion, oxygen saturation, mean arterial pressure, urine production, fluid administration), and surgery-related factors (duration, procedure, approach, vasopressor administration, epidural analgesia, additional procedures, excessive surgical field contamination, stoma creation).

All study outcomes were collected from electronic patient records and additionally reviewed by the research team. Complications were assessed based on final documentation by the lead surgeon and institutional complication registries. Additional baseline characteristics (age, sex, BMI, ASA grade, co-morbidities, intoxications, pathology results, distance from anal verge, neoadjuvant therapy, preoperative laboratory results) and details on the postoperative course (laboratory results, complications, length of stay, ICU stay, readmission, reinterventions, death) were collected at 90 days after surgery.

### Statistical analysis

Statistical analysis was conducted using SPSS^®^ (IBM, Armonk, NY, USA; version 28.0). A sample size calculation determined 1100 patients were required to observe an expected reduction in patients with ≥3 risk factors from 28.3% in previous research to ≤20% (α 0.05; β 0.80)^4^. However, to evaluate whether the observed outcomes aligned with the hypothesized improvements, an interim analysis was conducted. Patients with a maximum of one missing intraoperative risk factor were eligible for statistical analysis. Data are presented as *n* (%) when categorical and as mean(s.d.) or median (interquartile range) when continuous, depending on data skewness. Descriptive statistics (Pearson’s chi-squared test, Student’s *t* test, Mann–Whitney *U* test) were used to examine baseline characteristics and study outcomes. To evaluate change over time, outcomes from the first (Q1) and last (Q4) quartiles of the study interval were compared using similar descriptive statistics. Additionally, logistic regression analysis was performed to analyse the relationship between study groups, the intraoperative condition, and complications including CAL. Multivariate regression models were adjusted for significantly different baseline characteristics and hospital to control for potential confounders. Results are reported as OR (95% c.i.). *P* < 0.050 was considered statistically significant.

## Results

### Baseline

A total of 1256 patients were included in this study, of whom 372 (29.6%) were in the checklist-only group and 884 (70.4%) were in the care bundle group. Of the patients, 45.9% (577 of 1256) were female and the mean age of the patients was 69 (range 18–94) years. The indication for surgery was malignancy in 84.6% (996 of 1178) of the patients, with colonic resections performed in 86.5% (1086 of 1256) and rectal resections performed in 13.5% (170 of 1256). Overall, 1.4% (17 of 1256) of surgeries were initiated as open procedures and a diverting stoma was created in 3.9% (48 of 1256; 2.3% in colon and 13.9% in rectum).

In the checklist-only group, fewer patients were regular smokers (23.0% (72 of 313) *versus* 46.6% (408 of 876); *P* < 0.001) and regular alcohol consumers (6.5% (24 of 370) *versus* 54.8% (461 of 841); *P* < 0.001). Additionally, the duration of surgery (median of 177 (interquartile range 46) min *versus* median of 145 (interquartile range 63) min; *P* < 0.001) and the performance of additional resections (9.7% (36 of 372) *versus* 21.5% (186 of 864); *P* < 0.001) were significantly different. The intravenous fluid administration rate (median of 321 (interquartile range 157) ml/h *versus* median of 380 (interquartile range 248) ml/h; *P* < 0.001), the blood loss (median of 50 (interquartile range 35) ml *versus* median of 50 (interquartile range 50) ml; *P* < 0.001), and the mean(s.d.) arterial pressure (77(13) mmHg *versus* 84(16) mmHg; *P* < 0.001) were lower in the checklist-only group (*Table S1*).

### Intraoperative risk factors

**Fig. 2:**
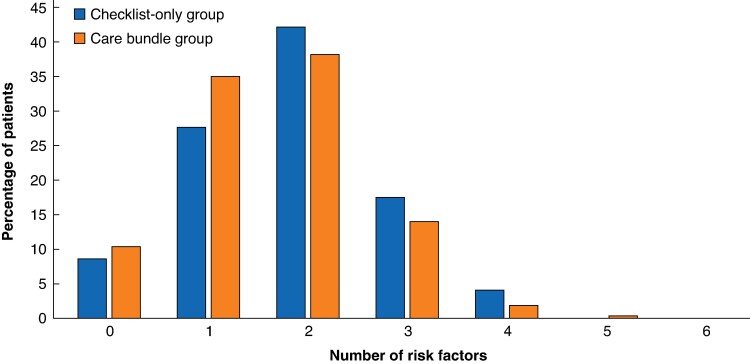
Intraoperative risk factors

The mean(s.d.) number of intraoperative risk factors present was 1.81(0.93) in the checklist-only group, compared with 1.63(0.96) in the care bundle group (*P* = 0.002) (*Fig. 2*). In the checklist-only group, 21.5% (80 of 372) of patients had ≥3 intraoperative risk factors, while this was 16.2% (143 of 884) in the care bundle group (*P* = 0.024).

### Overall postoperative complications

The overall postoperative complication rates differed significantly between the two groups (*P* < 0.001), with a rate of 26.6% (99 of 372) in checklist-only patients and a rate of 18.3% (162 of 884) in care bundle patients. Patients without complications were more likely to be female (47.4% (472 of 995) *versus* 40.2% (105 of 261); *P* = 0.038), had a lower ASA grade (median of II (interquartile range I) *versus* median of II (interquartile range I); *P* < 0.001), were less frequently diabetic (11.0% (106 of 967) *versus* 16.7% (43 of 258); *P* = 0.013), and less frequently received neoadjuvant therapy (7.1% (60 of 981) *versus* 14.4% (31 of 255); *P* < 0.001). Additionally, those patients had a shorter duration of surgery (median of 150 (interquartile range 65) min *versus* median of 162 (interquartile range 62) min; *P* < 0.001), less blood loss (median of 50 (interquartile range 50) ml *versus* median of 50 (interquartile range 79) ml; *P* < 0.001), and fewer diverting stomas (2.8% (28 of 994) *versus* 8.0% (21 of 261); *P* < 0.001) (*Table S2*).

### Anastomotic leakage

CAL was observed in 8.9% (33 of 372) of the checklist-only patients, compared with 6.2% (55 of 884) of the care bundle patients (*P* = 0.095). The severity of CAL was not significantly different between the two groups (*P* = 0.538). The median time until diagnosis was 6 (range 0–41) days and did not differ significantly between the two groups (*P* = 0.459).

Patients with CAL were younger (median of 67 (interquartile range 18) years *versus* median of 69 (interquartile range 17) years; *P* = 0.024) and more frequently received neoadjuvant therapy (18.9% (14 of 87) *versus* 7.8% (77 of 1151); *P* < 0.001). Additionally, those patients had a longer duration of surgery (median of 182 (interquartile range 79) min *versus* median of 150 (interquartile range 64) min; *P* < 0.001), had a lower intravenous fluid administration rate (median of 314 (interquartile range 169) ml/h *versus* median of 360 (interquartile range 230) ml/h; *P* = 0.010), and had more blood loss (median of 50 (interquartile range 50) ml and mean of 168 ml *versus* median of 50 (interquartile range 50) ml and mean of 97 ml; *P* = 0.047) (*Table S3*).

### Postoperative course

Other postoperative complications occurred in 15.5% (195 of 1256) of patients and differed significantly between the checklist-only group and the care bundle group (19.4% (72 of 372) *versus* 13.9% (123 of 884); *P* = 0.015) (*Table 2*). Complications included ileus in 3.7% (47 of 1256), delayed gastric emptying in 2.7% (34 of 1256), wound infection in 2.1% (26 of 1256), bleeding in 1.3% (16 of 1256), pneumonia in 1.3% (16 of 1256), urinary tract infection in 0.9% (11 of 1256), thromboembolic events in 0.1% (1 of 1256), atrial fibrillation in 1.9% (24 of 1256), and other unspecified complications in 4.5% (57 of 1256). The severity of these complications did not differ significantly between the two groups (*P* = 0.315).

**Table 2:** Postoperative course

Variable	Care bundle group (*n* = 884)	Checklist-only group (*n* = 372)	*P*
Postoperative complications	162 (18.3)	99 (26.6)	0.015*
**CAL**†	55 (6.2)	33 (8.9)	0.095
Grade A	1 (1.8)	0 (0.0)	0.538
Grade B	5 (9.1)	2 (6.1)	–
Grade C	49 (89.1)	31 (93.9)	–
**Other postoperative complications**‡	123 (13.9)	72 (19.4)	0.015*
Grade I	34 (27.6)	15 (20.5)	0.315
Grade II	54 (43.9)	37 (50.7)	–
Grade IIIa	12 (9.8)	4 (5.5)	–
Grade IIIb	15 (12.2)	9 (12.3)	–
Grade IV	3 (2.4)	6 (8.2)	–
Grade V	5 (4.1)	2 (2.7)	–
Length of stay (days), median (interquartile range)	4 (2)	3 (3)	0.235
ICU stay	27 (3.1)	19 (5.1)	0.078
Readmission	65 (7.4)	27 (7.3)	0.950
Mortality	7 (0.8)	6 (1.6)	0.211

Values are *n* (%) unless otherwise indicated. Other postoperative complications included: ileus, gastroparesis, wound infection, bleeding, pneumonia, urinary tract infection, thromboembolic events, atrial fibrillation, and other unspecified complications. *Statistically significant (*P* < 0.050). †Graded according to the ISREC classification. ‡Graded according to the Clavien–Dindo classification. CAL, colorectal anastomotic leakage; ISREC, International Study Group of Rectal Cancer.

Postoperative mortality was reported for 1.6% (6 of 372) of the checklist-only patients, compared with 0.8% (7 of 884) of the care bundle patients (*P* = 0.211). Length of stay was not different between the two groups (median of 3 (interquartile range 3) days *versus* median of 4 (interquartile range 2) days; *P* = 0.235). Additionally, rates of ICU stay (5.1% (19 of 372) *versus* 3.1% (27 of 884); *P* = 0.078) and readmission (7.3% (27 of 372) *versus* 7.4% (65 of 884); *P* = 0.950) were not significantly different.

### Impact over time

In the checklist-only group, between Q1 and Q4, no significant differences were observed in the number of intraoperative risk factors (1.75 *versus* 1.89; *P* = 0.351) or in the proportion of patients with ≥3 intraoperative risk factors (20.4% *versus* 22.6%; *P* = 0.721). Additionally, the rates of postoperative complications (25.8% *versus* 23.7%; *P* = 0.734) and CAL (9.7% *versus* 6.5%; *P* = 0.419) did not decrease significantly over time.

### Regression analysis

Overall complications were associated with checklist-only optimization (OR 1.49 (95% c.i. 1.08 to 2.05); *P* = 0.015). This association was subsequently adjusted for smoking, alcohol, duration of surgery, intravenous fluid administration, blood loss, mean arterial pressure, additional resection, sex, ASA grade, diabetes mellitus, neoadjuvant therapy, diverting stoma, and hospital. The multivariate regression analysis revealed a persistent significant relationship (OR 2.50 (95% c.i. 1.36 to 4.60); *P* = 0.003) (*Table 3*).

**Table 3:** Regression analysis

	*n* of *n* (%)	Univariate analysis		Multivariate analysis	
		OR (95% c.i.)	*P*	OR (95% c.i.)	*P*
**Postoperative complications**					
Care bundle	162 of 884 (18.3)	1.00	–	1.00	–
Checklist-only	99 of 372 (26.6)	1.49 (1.08,2.05)	0.015*	2.50 (1.36,4.60)†	0.003*
**CAL**					
Care bundle	55 of 884 (6.2)	1.00	–	1.00	–
Checklist-only	33 of 372 (8.9)	1.47 (0.94,2.30)	0.095	1.58 (0.95,2.62)‡	0.078

^*^Statistically significant (*P* < 0.050). †Corrected for: smoking, alcohol, duration of surgery, intravenous fluid administration, blood loss, mean arterial pressure, additional resection, sex, ASA grade, diabetes mellitus, neoadjuvant therapy, diverting stoma, and hospital. ‡Corrected for: smoking, alcohol, duration of surgery, intravenous fluid administration, blood loss, mean arterial pressure, additional resection, age, neoadjuvant therapy, and hospital. CAL, colorectal anastomotic leakage.

The occurrence of CAL was not significantly associated with checklist-only optimization (OR 1.47 (95% c.i. 0.94 to 2.30); *P* = 0.095). After correction for smoking, alcohol, duration of surgery, intravenous fluid administration, blood loss, mean arterial pressure, additional resection, age, neoadjuvant therapy, and hospital, this association remained non-significant (OR 1.58 (95% c.i. 0.95 to 2.62); *P* = 0.078).

## Discussion

This study demonstrated that optimization of the intraoperative condition in elective restorative colorectal surgery is not achieved through the use of an intraoperative checklist without combining it with supervision and feedback. Implementation of the enhanced care bundle was superior to the use of the intraoperative checklist alone, resulting in less exposure to modifiable CAL risk factors and fewer postoperative complications. The study highlights the importance of integrating the care bundle into local colorectal care pathways and implementing regular interim feedback on compliance to achieve sustainable improvements in intraoperative conditions for patients undergoing colorectal surgery.

The self-learning ability of surgical teams that is required when deploying checklist-only optimization was insufficient to improve postoperative outcomes. The results indicated that intraoperative conditions, as well as occurrence of postoperative complications and CAL, did not significantly change as the study progressed. Failure of the checklist-only optimization could be explained by research grounded in education and cognitive psychology, which suggests that constructive and continuous feedback significantly improves performance compared with self-learning alone^15–17^. Feedback facilitates faster error correction, prevents incorrect pattern reinforcement, and provides a sense of progress that can boost confidence and motivation^18^. Constructive feedback that is specific and actionable leads to greater improvements than generic feedback^19^. The EAGLE study demonstrated that adherence to educational modules for perioperative teams with regard to optimization strategies can reduce the incidence of postoperative complications^20^. While individuals directly involved in instances of CAL may develop strong self-learning skills due to their direct exposure to this dreadful complication, the risk of CAL is influenced by multiple factors and involves many healthcare providers. On a larger scale, external guidance and structured feedback are essential in supporting the entire care team by providing direction and consistency. Additionally, studies suggest that incorporating incentives, such as economic or personal rewards, can further enhance motivation to comply with such initiatives^21,22^.

Due to the clinically and statistically significant higher occurrence of complications in the checklist-only group, further patient inclusions were discontinued. Although no statistically significant difference in CAL was observed (*P* = 0.095), the clinical difference between a CAL rate of 6.2% and 8.9% remains noteworthy, as this corresponds with a 43.5% increase of this potentially life-threatening complication. This is particularly relevant given that patients in the checklist-only group experienced more favourable conditions regarding exposure to well-established fixed/non-modifiable risk factors (for example fewer rectal resections, surgeries with fewer intraoperative events, less blood loss)^23,24^.

Key strengths of this study include its prospective and multicentre design, which enhances the generalizability of the findings. Additionally, the intraoperative checklist is simple to implement and not time-consuming, making it highly feasible for global clinical practice. Furthermore, the interventions within the enhanced care bundle are widely accessible and relatively inexpensive, further supporting their applicability in routine surgical care. This interventional study is the first to evaluate the quality of implementation not only during the study interval but also in real-world clinical settings, ensuring its relevance beyond controlled research conditions. A key point related to this is that this study included a broad range of patients, encompassing both colonic and rectal resections, as well as operations where a diverting stoma was constructed. Optimizing intraoperative conditions and consequently preventing CAL may benefit all patients, regardless of the specific surgical procedure performed.

The present study has several limitations, including its non-randomized design. However, the risk of bias was minimized through multivariate regression analysis and by including clinically similar study groups to ensure comparability. Allocation to study groups occurred at hospital level rather than at patient level, as this was considered the only feasible approach in clinical practice. The analyses were adjusted for the individual hospital in the multivariate regression to account for potential hospital-level differences. Moreover, the study was fully unblinded, which increases the risk of bias. The majority of participating hospitals were Dutch, potentially limiting the validity to all surgical practices worldwide. Additionally, other safety procedures were not standardized, and may have varied over time and across hospitals, and compliance with ERAS guidelines was not monitored. Information regarding the nuances of intraoperative decision-making and individual surgical experience were not collected. Nonetheless, statistical corrections were applied to account for these variations and mitigate potential confounding effects.

In conclusion, this study demonstrated that integration of the enhanced care bundle into local colorectal care pathways combined with regular interim feedback on compliance was associated with improved intraoperative conditions and better postoperative outcomes in patients undergoing elective colorectal surgery involving creation of an anastomosis. While these findings are encouraging, a randomized study is needed to strengthen generalizability, possibly additionally benefiting from incorporating (digital) mechanisms to support sustained adherence. Future investments in structured and guided implementation of care bundles, along with ongoing feedback, should be prioritized to ensue optimal performance in real-world clinical practice.

## Collaborators

Anne de Wit (Department of Surgery, Amsterdam University Medical Centre, Amsterdam, The Netherlands; and Cancer Centre Amsterdam, Amsterdam, The Netherlands); Daitlin E. Huisman (Department of Surgery, Amsterdam University Medical Centre, Amsterdam, The Netherlands; and Cancer Centre Amsterdam, Amsterdam, The Netherlands); Boukje T. Bootsma (Department of Surgery, Amsterdam University Medical Centre, Amsterdam, The Netherlands; and Cancer Centre Amsterdam, Amsterdam, The Netherlands); Geert Kazemier (Department of Surgery, Amsterdam University Medical Centre, Amsterdam, The Netherlands; and Cancer Centre Amsterdam, Amsterdam, The Netherlands); Jennifer M. J. Schreinemakers (Department of Surgery, Amphia Ziekenhuis, Breda, The Netherlands); Bas Frietman (Department of Surgery, Maasziekenhuis—Pantein, Boxmeer, The Netherlands); Lindsey de Nes (Department of Surgery, Maasziekenhuis—Pantein, Boxmeer, The Netherlands); Freek Daams (Department of Surgery, Amsterdam University Medical Centre, Amsterdam, The Netherlands; and Cancer Centre Amsterdam, Amsterdam, The Netherlands).

## Supplementary Material

znaf236_Supplementary_Data

## Data Availability

Deidentified individual participant data collected in the SmartCheck study and additional relevant related documents can be made available upon request by contacting A.d.W. (a.dewit1@amsterdamumc.nl) or F.D. (f.daams@amsterdamumc.nl). There are no date restrictions on the availability of the data. The SmartCheck investigators will be allowed to approve all research performed with the shared data.
