# Supplementary material for: Optimizing intraoperative conditions in patients undergoing elective colorectal surgery to prevent anastomotic leakage: SmartCheck study
Source: Br J Surg. 2025 Dec 1;112(12):znaf236. doi: 10.1093/bjs/znaf236 (PMC12667027; doi:10.1093/bjs/znaf236)
Supplement: znaf236_Supplementary_Data [file znaf236_supplementary_data.docx]

**Optimizing intraoperative conditions in patients undergoing elective colorectal surgery to prevent anastomotic leakage – the SmartCheck study**

By the Taskforce Anastomotic Leakage

*Correspondence address*

F. Daams, MD PhD

Department of Surgery, Amsterdam University Medical Center, Cancer Center Amsterdam

De Boelelaan 1117, 1081HH Amsterdam, the Netherlands, Room ZH-7F

E: [f.daams@amsterdamumc.nl](mailto:f.daams@amsterdamumc.nl) P: +31655688542

**Supplementary Materials - Index**

| **Supplementary Figures and Tables** |  |
| --- | --- |
| Table S1: Baseline characteristics by study groups | *page 2* |
| Table S2: Baseline characteristics by postoperative complications | *page 3* |
| Table S3: Baseline characteristics by colorectal anastomotic leakage | *page 4* |

**Supplementary Figures and Tables**

Table S1: Baseline characteristics by study group

|  | **Care bundle (n=884)** | | **Checklist-only (n=372)** | |  |
| --- | --- | --- | --- | --- | --- |
| **Variable** |  | **Missing (n=)** |  | **Missing (n=)** | **p value** |
| Age (years) | 68 (17) |  | 68 (18) | 1 | 0.767 |
| Sex (female) | 402 (45.5%) |  | 175 (47.0%) |  | 0.611 |
| BMI (kg/m^2^) | 25.7 (5.4) | 15 | 26.1 (6.3) | 1 | 0.258 |
| ASA-classification | 2 (1) | 49 | 2 (1) | 1 | 0.781 |
| Diabetes mellitus (yes) | 104 (12.2%) | 31 | 45 (12.1%) |  | 0.962 |
| History of smoking (yes) | 408 (46.6%) | 8 | 72 (23.0%) | 59 | **<0.001** |
| Alcohol usage (yes) | 461 (54.8%) | 43 | 24 (6.5%) | 2 | **<0.001** |
| Diagnosis (malign) | 685 (85.0%) | 78 | 311 (83.6%) |  | 0.541 |
| Neoadjuvant therapy (yes) | 72 (9.7%) | 18 | 19 (6.1%) |  | 0.061 |
| Chemotherapy | 19 (26.4%) |  | 9 (47.4%) |  |  |
| Radiotherapy | 28 (38.9%) |  | 3 (15.8%) |  |  |
| Chemo-radiotherapy | 25 (34.7%) |  | 4 (21.1%) |  |  |
| Immunotherapy | 0 |  | 3 (15.8%) |  |  |
| Surgical procedure |  |  |  |  | **<0.001** |
| Ileocoecal resection | 19 (2.1%) |  | 21 (5.6%) |  |  |
| Right-sided hemicolectomy | 352 (39.8%) |  | 136 (36.6%) |  |  |
| Transversum resection | 23 (2.6%) |  | 18 (4.8%) |  |  |
| Left-sided hemicolectomy | 78 (8.8%) |  | 25 (6.7%) |  |  |
| Sigmoidectomy | 233 (26.4%) |  | 146 (39.2%) |  |  |
| Rectum resection | 153 (17.3%) |  | 17 (4.6%) |  |  |
| Hartmann reversal | 17 (1.9%) |  | 2 (0.5%) |  |  |
| Segment resection | 1 (0.1%) |  | 0 |  |  |
| Subtotal colectomy | 6 (0.7%) |  | 7 (1.9%) |  |  |
| Other | 2 (0.2%) |  | 0 |  |  |
| Surgery duration (minutes) | 145 (63) | 22 | 177 (46) |  | **<0.001** |
| Surgical approach (open) | 14 (1.6%) |  | 3 (0.8%) |  | 0.276 |
| Deviating stoma (yes) | 31 (3.5%) | 1 | 18 (4.8%) |  | 0.267 |
| Additional organ resection (yes) | 186 (21.5%) | 20 | 36 (9.7%) |  | **<0.001** |
| Blood loss (ml) | 50 (50) | 23 | 50 (35) | 1 | **<0.001** |
| MAP (mmHg) | 84 (16) | 18 | 77 (13) | 10 | **<0.001** |
| Fluids administration (ml/hour) | 380 (248) | 30 | 321 (157) |  | **<0.001** |
| Data is presented as number (%) or medians (interquartile range).  A p value <0.05 was considered statistically significant and marked bold.  BMI: body mass index; ASA: American Society of Anesthesiology; MAP: Mean Arterial Pressure. | | | | | |

Table S2: Baseline characteristics by postoperative complications

|  | **Uncomplicated (n=995)** | | **Complicated (n=261)** | |  |
| --- | --- | --- | --- | --- | --- |
| **Variable** |  | **Missing (n=)** |  | **Missing (n=)** | **p value** |
| Age (years) | 67 (17) |  | 72 (18) | 1 | 0.113 |
| Sex (female) | 472 (47.4%) |  | 105 (40.2%) |  | **0.038** |
| BMI (kg/m^2^) | 25.8 (5.6) | 16 | 25.5 (6.3) |  | 0.528 |
| ASA-classification | 2 (1) | 45 | 2 (1) | 4 | **<0.001** |
| Diabetes mellitus (yes) | 106 (11.0%) | 28 | 43 (16.7%) | 3 | **0.013** |
| History of smoking (yes) | 373 (39.5%) | 51 | 107 (43.7%) | 16 | 0.237 |
| Alcohol usage (yes) | 396 (41.3%) | 35 | 89 (35.5%) | 10 | 0.095 |
| Diagnosis (malign) | 790 (84.9%) | 65 | 206 (83.1%) | 13 | 0.466 |
| Neoadjuvant therapy (yes) | 60 (7.1%) | 14 | 31 (14.4%) | 5 | **<0.001** |
| Chemotherapy | 22 (36.7%) |  | 6 (19.4%) |  |  |
| Radiotherapy | 17 (28.3%) |  | 14 (45.2%) |  |  |
| Chemo-radiotherapy | 19 (31.7%) |  | 10 (32.3%) |  |  |
| Immunotherapy | 2 (3.3%) |  | 1 (3.2%) |  |  |
| Surgical procedure |  |  |  |  | 0.105 |
| Ileocoecal resection | 28 (2.8%) |  | 12 (4.6%) |  |  |
| Right-sided hemicolectomy | 384 (38.6%) |  | 104 (39.8%) |  |  |
| Transversum resection | 29 (2.9%) |  | 12 (4.6%) |  |  |
| Left-sided hemicolectomy | 84 (8.4%) |  | 19 (7.3%) |  |  |
| Sigmoidectomy | 317 (31.9%) |  | 62 (23.8%) |  |  |
| Rectum resection | 129 (13.0%) |  | 41 (15.7%) |  |  |
| Hartmann reversal | 12 (1.2%) |  | 7 (2.7%) |  |  |
| Segment resection | 1 (0.1%) |  | 0 |  |  |
| Subtotal colectomy | 9 (0.9%) |  | 4 (1.5%) |  |  |
| Other | 2 (0.2%) |  | 0 |  |  |
| Surgery duration (minutes) | 150 (65) | 20 | 162 (62) | 2 | **<0.001** |
| Surgical approach (open) | 12 (1.2%) |  | 5 (1.9%) |  | 0.398 |
| Deviating stoma (yes) | 28 (2.8%) | 1 | 21 (8.0%) |  | **<0.001** |
| Additional organ resection (yes) | 170 (17.4%) | 20 | 52 (19.9%) |  | 0.352 |
| Blood loss (ml) | 50 (50) | 23 | 50 (79) | 1 | **<0.001** |
| MAP (mmHg) | 83 (16) | 25 | 81 (16) | 3 | 0.095 |
| Fluids administration (ml/hour) | 349 (217) | 28 | 371 (264) | 2 | 0.793 |
| Data is presented as number (%) or medians (interquartile range).  A p value <0.05 was considered statistically significant and marked bold.  BMI: body mass index; ASA: American Society of Anesthesiology; MAP: Mean Arterial Pressure. | | | | | |

Table S3: Baseline characteristics by colorectal anastomotic leakage

|  | **No CAL (n=1168)** | | **CAL (n=88)** | |  |
| --- | --- | --- | --- | --- | --- |
| **Variable** |  | **Missing (n=)** |  | **Missing (n=)** | **p value** |
| Age (years) | 69 (17) | 1 | 67 (18) |  | **0.024** |
| Sex (female) | 539 (46.1%) |  | 38 (43.2%) |  | 0.590 |
| BMI (kg/m^2^) | 25.7 (5.8) | 14 | 26.3 (5.7) | 2 | 0.415 |
| ASA-classification | 2 (1) | 45 | 2 (1) | 4 | 0.164 |
| Diabetes mellitus (yes) | 138 (12.1%) | 30 | 11 (12.6%) | 1 | 0.887 |
| History of smoking (yes) | 442 (40.0%) | 63 | 38 (45.2%) | 4 | 0.346 |
| Alcohol usage (yes) | 454 (40.4%) | 43 | 31 (36.0%) | 2 | 0.432 |
| Diagnosis (malign) | 926 (84.6%) | 73 | 70 (84.3%) | 3 | 0.956 |
| Neoadjuvant therapy (yes) | 77 (7.8%) | 17 | 14 (18.9%) | 1 | **0.001** |
| Chemotherapy | 28 (36.4%) |  | 0 |  |  |
| Radiotherapy | 22 (28.6%) |  | 9 (64.3%) |  |  |
| Chemo-radiotherapy | 24 (31.2%) |  | 5 (35.7%) |  |  |
| Immunotherapy | 3 (3.9%) |  | 0 |  |  |
| Surgical procedure |  |  |  |  | **0.032** |
| Ileocoecal resection | 38 (3.3%) |  | 2 (2.3%) |  |  |
| Right-sided hemicolectomy | 467 (40.0%) |  | 21 (23.9%) |  |  |
| Transversum resection | 39 (3.3%) |  | 2 (2.3%) |  |  |
| Left-sided hemicolectomy | 97 (8.3%) |  | 6 (6.8%) |  |  |
| Sigmoidectomy | 349 (29.9%) |  | 30 (34.1%) |  |  |
| Rectum resection | 148 (12.7%) |  | 22 (25.0%) |  |  |
| Hartmann reversal | 16 (1.4%) |  | 3 (3.4%) |  |  |
| Segment resection | 1 (0.1%) |  | 0 |  |  |
| Subtotal colectomy | 11 (0.9%) |  | 2 (2.3%) |  |  |
| Other | 2 (0.2%) |  | 0 |  |  |
| Surgery duration (minutes) | 150 (64) | 22 | 182 (79) |  | **<0.001** |
| Surgical approach (open) | 16 (1.4%) |  | 1 (1.1%) |  | 0.851 |
| Deviating stoma (yes) | 45 (3.9%) | 1 | 4 (4.5%) |  | 0.753 |
| Additional organ resection (yes) | 205 (17.9%) | 20 | 17 (19.3%) |  | 0.713 |
| Blood loss (ml) | 50 (50) | 22 | 50 (50) | 2 | **0.047** |
| MAP (mmHg) | 83 (15) | 27 | 81 (13) | 1 | 0.830 |
| Fluids administration (ml/hour) | 360 (230) | 30 | 314 (169) |  | **0.010** |
| Data is presented as number (%) or medians (interquartile range).  A p value <0.05 was considered statistically significant and marked bold.  CAL: Colorectal Anastomotic Leakage; BMI: body mass index; ASA: American Society of Anesthesiology; MAP: Mean Arterial Pressure. | | | | | |
